# Jack pine’s responses to climate change: increased water–use efficiency but evident growth limitations in dry environments

**DOI:** 10.1093/treephys/tpaf102

**Published:** 2025-08-22

**Authors:** Oloruntobi Gideon Olugbadieye, Etienne Boucher, Annie Deslauriers, Yves Bergeron, Eric Rosa, Marc-André Lemay, Fabio Gennaretti

**Affiliations:** Groupe de Recherche en Écologie de la MRC Abitibi, Institut de Recherche sur les Forêts, Université du Québec en Abitibi-Témiscamingue, 341, Rue Principale Nord, Amos, Québec J9T 2L8, Canada; Department of Geography, GEOTOP and Centre d’études Nordiques, Université du Québec à Montréal, 1255, St - Denis, Montréal, Québec H2X 3R9, Canada; Departement des Sciences Fondamentales, Université du Québec à Chicoutimi, 555, Boulevard de l’Université, Chicoutimi, Québec G7H 2B1, Canada; Groupe de Recherche en Écologie de la MRC Abitibi, Institut de Recherche sur les Forêts, Université du Québec en Abitibi-Témiscamingue, 341, Rue Principale Nord, Amos, Québec J9T 2L8, Canada; Département des sciences biologiques, Université du Québec à Montréal, Case Postale 8888, Succursale Centre-Ville, Montréal, Québec H3C 3P8, Canada; Groundwater Research Group, Research Institute on Mines and Environment (RIME), Université du Québec en Abitibi-Témiscamingue (UQAT), 341, Rue Principale Nord, Amos, Québec J9T 2L8, Canada; Groupe de Recherche en Écologie de la MRC Abitibi, Institut de Recherche sur les Forêts, Université du Québec en Abitibi-Témiscamingue, 341, Rue Principale Nord, Amos, Québec J9T 2L8, Canada; Groupe de Recherche en Écologie de la MRC Abitibi, Institut de Recherche sur les Forêts, Université du Québec en Abitibi-Témiscamingue, 341, Rue Principale Nord, Amos, Québec J9T 2L8, Canada; Department of Agricultural, Food and Environmental Sciences, Università Politecnica delle Marche, Via Brecce Bianche 10, Ancona 60131, Italy

**Keywords:** carbon isotopes, iWUE, oxygen isotopes, stomata conductance, tree rings

## Abstract

*Pinus banksiana* Lamb. exhibits remarkable ecological adaptability, thriving across diverse environments in the Canadian boreal zone, including clay deposits, fast-draining glacial tills and rocky outcrops. However, projected rising temperature and increasing vapor pressure deficit (VPD) could increase the species’ vulnerability, particularly in dry regions. In this study, we measured basal area increment (BAI) and physiological responses from isotopic fractionation across a soil gradient including three sites in the boreal mixed wood of western Quebec, Canada. The sites were a clay-rich soil (CLY, a humid site), an esker base (ESB, an intermediate site) and an esker top (EST, a sandy, well drained, dry site). Using tree-ring analysis and dual stable isotopes (δ^13^C and δ^18^O), we evaluated intrinsic water-use efficiency (iWUE) and leaf water enrichment (Δ^18^O_lw_). Our results revealed a significant correlation between Δ^18^O_lw_ and VPD, indicating that stomatal regulation is the crucial physiological mechanism controlling *P. banksiana*’s response to environmental stress across the sites. This effect was most pronounced at the dry EST site, where higher iWUE and less negative δ^13^C values suggest greater stomatal limitation of CO_2_ uptake. Increased iWUE was associated with enhanced BAI in the humid CLY site and a negative iWUE–BAI relationship emerged at EST, suggesting carbon assimilation constraints under drier conditions. Our results reveal a physiological trade-off in *P. banksiana* across a soil moisture gradient, demonstrating that rising atmospheric demand may decouple water-use efficiency from growth in drier environments like the EST site. By integrating isotopic signatures with growth dynamics, our study identifies a potential ecological tipping point beyond which increased iWUE may no longer sustain carbon gain under intensifying climate stress.

## Introduction

Intrinsic water-use efficiency (iWUE), defined as the ratio of carbon assimilation rate by photosynthesis (*A*) to stomatal conductance (g_s_), represents a key physiological parameter for trees and other plants. It serves as a key indicator of stomatal responses and carbon uptake under changing conditions (Kannenberg et al. 2021, Ren et al. 2022, Weiwei et al. 2018). Tree iWUE is sensitive to the rise in atmospheric CO₂ concentration (C_a_) since the Industrial Revolution and to climate variations. However, the physiological mechanisms driving iWUE changes and its general increasing trend over time are species-specific and site-specific (Guerrieri et al. 2019; Peñuelas et al. 2011; Pu and Lyu 2022). A rise in iWUE can result from a reduction in g_s_, an increase in *A* or a combination of both (Guerrieri et al. 2019; Liu et al. 2014*b*; Martínez-Sancho et al. 2018). Understanding these physiological mechanisms and environmental drivers is crucial for predicting forest productivity and carbon cycle dynamics.

Over the past two decades, tree-ring stable isotopes, particularly the carbon (δ^13^C) and oxygen (δ^18^O) isotopic compositions, have become valuable proxies for understanding iWUE changes in response to rising C_a_ and to climate variations over seasonal to decadal timescales (Battipaglia et al. 2013; Lévesque et al. 2014). On one hand, δ^13^C provides direct insights into iWUE by reflecting the balance between photosynthesis and stomata conductance. During photosynthesis, when stomata are open, plants preferentially assimilate ^12^C over ^13^C, resulting in a discrimination against ^13^C. This stable carbon isotope discrimination (Δ^13^C) is influenced by water stress and g_s_. Under soil water stress and high vapor pressure deficit (VPD), plant reduced g_s_ to conserve water, which in turn limits CO_2_ diffusion into leaves, reducing discrimination against ^13^C and resulting in higher δ^13^C value in photosynthetic assimilates. These assimilates, are ultimately transported to the stem and incorporated into cellulose during wood formation, thus keeping track of the δ^13^C leaf signal, which is commonly used to infer iWUE (Farquhar et al. 1982, 1989*a*; Ma et al. 2023).

On the other hand, δ^18^O in tree-rings offers valuable insights into evaporative demand at leaf level and water availability, which influence stomata regulation and consequently contribute to variations in iWUE (Battipaglia et al. 2013; Gómez-Guerrero et al. 2013). Under water stress, g_s_ tends to decline, reducing water loss through transpiration. This leads to a longer residence time of water within the leaf, which enhances the evaporative enrichment of ^18^O in leaf water. These enriched leaf water signals are incorporated into sugars during photosynthesis and later fixed into stem cellulose during wood formation, thereby partially preserving a record of the environmental condition influencing g_s_ (Barbour & Farquhar, 2000; Gessler et al. 2014; McCarroll & Loader, 2004; Yakir 1992). δ^18^O in tree rings is independent of variations in *A* but is closely linked to changes in leaf water dynamics and g_s_, often showing negative correlation with g_s_ due to reduced transpiration under low humidity or water stress (Scheidegger et al. 2000; Siegwolf et al. 2023).

Combined analyses of δ^13^C and δ^18^O in tree rings are used to determine changes in *A* and g_s_ in response to elevated CO_2_ and climate variability (Lévesque et al. 2014; Liu et al. 2014*a*; McCarroll & Loader, 2004; Scheidegger et al. 2000; Siegwolf et al. 2023). Recently, integrated use of δ^13^C and δ^18^O in tree rings has become widely applied in studies evaluating tree iWUE responses (Guerrieri et al. 2019; Mathias & Thomas, 2021; Palandrani et al. 2021; Pu & Lyu, 2022). Such a dual-isotope approach has the potential to elucidate site-specific vulnerabilities of tree species, particularly in relation to soil type, drainage and water availability.

The boreal forests of western Quebec contain a diverse array of surficial glacial soil deposits, including till deposits, glaciofluvial formations (eskers and moraines) and clay deposits that have accumulated during the last glaciation-deglaciation cycle (Veillette 1994, Ritchie 2004). These contrasting soil conditions can significantly influence tree growth and their responses to changing climates. For example, Royer-Tardif & Bradley (2011) found that the most fertile clay deposits promote heterogeneous forest compositions and foster tree growth. Similarly, Chavardès et al. (2022) highlight that sandy soils, with rapid drainage, limit tree growth. Indeed, soils with excessive stoniness and sandy textures often exhibit rapid drainage (Bernier et al. 2006) and are typically less productive for tree growth (Béland & Bergeron, 1996). Despite these contrasting soil conditions, *Pinus banksiana* Lamb. (jack pine) one of the dominant and most commercially valuable boreal tree species, providing essential pulpwood, lumber and round timber, demonstrate remarkable ecological adaptability, enabling them to grow on several surficial deposits (Bergeron & Bouchard, 1984). Comparing *P. banksiana* physiology on fast-draining surficial deposits with its response on other sites would be particularly valuable, as these sites can serve as sentinels for future climate change impacts. Furthermore, while studies have explored how boreal tree species respond on different soil types in terms of growth (Hofgaard et al. 1999; Lecomte & Bergeron, 2005), the underlying physiological mechanisms driving these responses remain misunderstood. Understanding these responses is key to predicting how forests will adapt with climate change.

In this study, we examined the responses of *P. banksiana* to changing climate conditions across contrasting soil types. Using tree-ring α-cellulose δ^13^C and δ^18^O, we documented 33-year trends (1992–2022) in iWUE for *P. banksiana* on different soil types: clay soil and sandy soils on esker. We estimated changes in iWUE using δ^13^C and assessed δ^18^O to determine the enrichment of ^18^O in leaf water above source water (Δ^18^O_lw_). While Δ^18^O_lw_ reflects variability in transpiration and g_s_ alone, δ^13^C captures combined changes due to *A* and g_s_. Photosynthetic carbon gain was also contextualized through an analysis of basal area increment (BAI). The main objectives of this study were to: (i) examine iWUE, *A*, g_s_ and growth of *P. banksiana* across different soil types, specifically sandy and clay soils, and (ii) determine the environmental factors influencing iWUE and BAI in *P. banksiana* across these soil types. We hypothesize that g_s_ would be the primary physiological mechanism regulating iWUE across our study sites. However, we expect its influence to be more pronounced in drier environments where stronger stomatal regulation reduces transpiration. This increase in iWUE, however, is anticipated to come at the cost of reduced tree growth, as stomatal closure limits carbon assimilation. If this hypothesis is verified, the study will provide a solid foundation for predicting the critical impacts of climate change on the future growth of *P. banksiana*.

## Materials and methods

### Study area and climate

**Figure 1:**
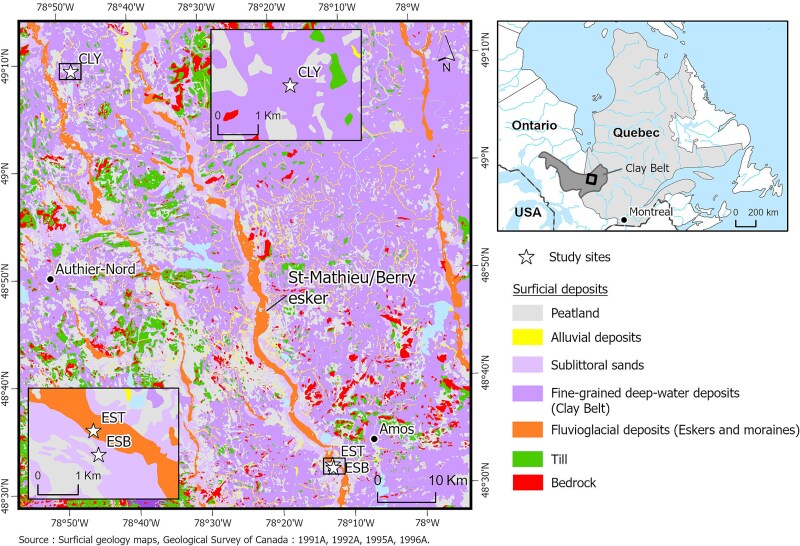
Map of the study area showing the locations of the three study sites (CLY, ESB and EST). The main map depicts the broader study region with a 10 km scale bar for regional context. The inset map (labeled CLY at the top and EST, ESB at the bottom left) zooms into the specific study area and uses a 1 km scale bar to show finer spatial detail within the main map.

The study area spans from latitude 48.31° to 49.11° N and longitude 78.12° to 78.51° W in the Abitibi region of western Québec, Canada (Figure 1). It falls within the fir-white birch bioclimatic domain and the southern portion of the black spruce-moss bioclimatic domain (Saucier et al. 2011). We established three forested sites within the study area: one on clay soil (CLY) in Authier-Nord and two on sandy soil (ESB—Esker Base, and EST—Esker top) in Amos. These three sites form a gradient of soil moisture conditions due to the nature of surficial deposits and topography, ranging from the more humid CLY to the drier EST. The clay site (CLY) was located at an elevation of 311 m above sea level (a.s.l.) and has a mid-summer average water table depth of ~0–5 m (data obtained from the nearby points of a map derived from interpolation of piezometric data; Cloutier et al. 2015). In early spring, the water table is close to the soil surface and remains at this level until late May.

The sandy soil sites in Amos (ESB and EST) were located on the fluvioglacial deposits of the Saint-Mathieu/Berry esker. ESB was located at the base of the esker, in proximity to the clay plain deposits at an elevation of 324 m a.s.l. and had an average water table depth between 5–10 m (Cloutier et al. 2015). This site was also located ~50 m away from a peatland next to the esker. In contrast, EST was located at the top of the esker at an elevation of 342 m a.s.l., 1 km west of ESB. This site was characterized by a predominantly dry environment, with a stable water table depth ranging between 20 and 25 meters (Cloutier et al. 2015). Infiltration and recharge processes at EST occur at a rapid rate.

**Figure 2:**
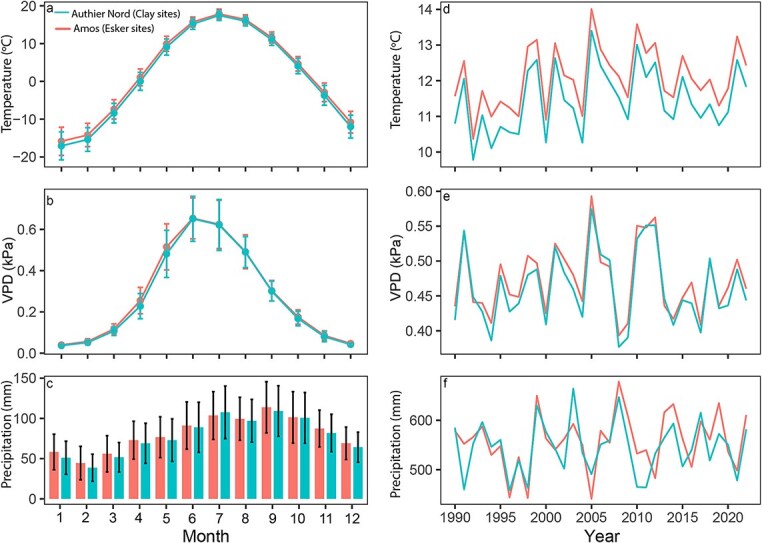
Climatic records for Amos (ESB and EST) and Authier Nord (CLY): (a–c) multi-year (1990–2022) monthly averages ± standard deviation as error bars of temperature, total precipitation and VPD; (d–f) growing season mean temperature, total precipitation and mean VPD from 1990 to 2022. Data obtained from the Copernicus Climate Change Service (2024).

The gradient in water availability across sites was further evidenced by variations in vegetation. The forest stand on CLY site consists of a mixed wood which includes both coniferous and deciduous species, such as trembling aspen (*Populus tremuloides* Michx), jack pine (*Pinus banksiana* Lamb), white birch (*Betula papyrifera* Marshall), and black spruce (*Picea mariana* (Mill.) B.S.P.). The esker sites ESB and EST consist mostly of coniferous species, specifically black spruce and jack pine. However, the ESB site were mostly dominated by black spruce, while the EST site was mainly dominated by jack pine. The abundance of jack pine at the EST site can be attributed to its ability to grow in well-drained, sandy, and nutrient-poor soil, as well as its flexible rooting system that can penetrate deep into the soil to access water and nutrients (Burns, 1990). The study sites experience mild, humid summers and long, cold winters. At the Authier-Nord site, the mean growing season (April–September) temperature is 11.5 °C with a mean growing season precipitation of 544.7 mm (1990–2022; Figure 2). At the Amos sites (ESB and EST), the mean growing season temperature is 12.1 °C and mean growing season precipitation was 557.6 mm (1990–2022; Figure 2). Climate data was obtained from ERA5 Copernicus Climate Change Service (Muñoz-Sabater et al. 2021). ERA5 has been proved to be valuable for dendro-climatology analyses (Wang et al. 2024). A summary of the study site characteristics is provided in Table S1 available as Supplementary Data at *Tree Physiology* Online.

### Tree sampling and dendrochronological analysis

Tree-ring cores were collected from five healthy jack pine trees in each of the three study sites using a 1.2 cm increment borer. Diameter at breast height of trees was similar across sites ranging from 26 to 31 cm. Two cores per tree were taken at breast height (1.3 m) from two opposite directions of each tree. The adequacy of this sampling strategy has been supported by recent studies employing similar sample sizes in dendrochronological research (Benisiewicz et al. 2024; Llanos-Campana et al. 2025). Tree-ring analyses were processed using standard dendrochronological techniques (Speer, 2010). All tree rings were dated from bark to pith. Tree-ring widths were measured using Coorecorder software v. 9.8.0 (Cybis Elektronik & Data AB, Saltsjöbaden, Sweden). Cross-dating was later verified using PAST5 (version 5.0.620, SCIEM, Vienna, Austria). We derived series of yearly BAI from raw ring widths (Figure S3 available as Supplementary Data at *Tree Physiology* Online) assuming circular tree rings and estimating distance to pith if needed with Coorecorder. BAI were used instead of ring-width indexes because BAI is less dependent on age after juvenile growth and thus avoids the need for additional detrending (Biondi 1999; Klesse & Bigler, 2025), with the calculation performed in R using dplR library (Bunn, 2008). The summarized information about tree-ring chronologies were presented in Table S2 available as Supplementary Data at *Tree Physiology* Online.

### Stable oxygen and carbon isotope analysis

For stable isotope analyses, we focused on tree rings formed between 1990 to 2022 to minimize the influence of the ‘juvenile effect’ which can bias isotopic measurements in the early stages of tree growth (Leavitt, 2010). Each annual ring in the 1990–2022 interval was separated under a binocular microscope using a thin sharp blade. The corresponding rings were carefully pooled to create a single annual isotope series per site, ensuring equal mass contribution from each tree. However, to evaluate the spread of individual isotopic values, the tree rings for the years 1990, 2000, 2010 and 2020 were not pooled and were measured for each tree. Each wood sample (pooled and unpooled) was ground into fine particles using a Retsch MM400 Mixer Mill. To minimize the risk of contamination by plastic particles (Isaac-Renton et al. 2016), we used stainless steel balls and vials for grounding. We then extracted of the α-cellulose according to the protocol of Namvar et al. (2024). Approximately 0.4–0.5 mg of the α-cellulose was loaded into tin foil capsules and combusted for δ^13^C analysis using a Micromass Model Isoprime 100 Isotope Ratio Mass Spectrometer (IRMS) coupled with an Elementar Vario MicroCube elemental analyzer. The analytical precision of δ^13^C was better than ±0.1‰ (standard deviation). For δ^18^O analysis, around 0.3–0.4 mg of α-cellulose was weighed into silver cups to standardize mass amounts across samples. The analysis was performed using an Isoprime Vision isotope ratio mass spectrometer coupled to an Elementar Vario PyroCube elemental analyzer in continuous flow mode. Results were normalized using two internal references on the VSMOW-SLAP scale, and a third reference was tested as an unknown to verify the accuracy of the process. The total analytical uncertainty was within ±0.3‰. Both δ^13^C and δ^18^O values are reported in parts per thousand (per mille, ‰), relative to the Vienna Pee Dee Belemnite (VPDB) for carbon ratios and Vienna Standard Mean Ocean Water (VSMOW) for oxygen ratios. All isotope measurements were conducted at the Stable Isotope Laboratory of the Geotop-Université du Québec à Montréal research centre in Montreal, Canada.

### Estimates derived from carbon isotope values

We corrected the measured carbon isotope values to a pre-industrial standard value of −6.4‰, following the method described by McCarroll et al. (2009) using Eq. (1). This correction was necessary due to the declining trend observed in δ^13^C values during the industrial period, which was caused by the burning of fossil fuels, also known as the ‘Suess effect.’


(1) \begin{equation*} {\delta}^{13}{C}_{cor}={\delta}^{13}{C}_{plant}-\left({\delta}^{13}{C}_{atm}-\Big(-6.4\right)\Big) \end{equation*}

Where ${\delta}^{13}{C}_{plant}$ is measured tree ring isotope composition, ${\delta}^{13}{C}_{atm}$ is the annual value of the isotopic composition of atmospheric CO_2_ and −6.4‰ is pre-industrial standard value. We compiled the historic δ^13^C value of atmospheric CO_2_ from the studies of Belmecheri & Lavergne (2020) and Mathias & Hudiburg (2022).

According to Farquhar et al. (1982), the stable isotope discrimination (∆^13^C) in C_3_ plants against ^13^C indicates the isotope changes between the atmosphere and plants as expressed by:


(2) \begin{equation*} {\varDelta}^{13}C=\frac{\left({\delta}^{13}{C}_{atm}-{\delta}^{13}{C}_{cor}\right)}{\left(1+\frac{\delta^{13}{C}_{cor}}{1000}\right)}=a+\left(b-a\right)\frac{C_i}{C_a} \end{equation*}

Where *a* (~4.4‰) is the isotopic fractionation coefficient of CO_2_ diffusion from the atmosphere into the intercellular space of mesophyll cells, *b* (~27‰) is the discrimination value for carboxylation, and *C_i_* and *C_a_* are the intercellular and atmospheric C_a_, respectively. Considering Eq. 2, Δ^13^C can be converted to *C_i_/C_a_* ratios according to the following equation Farquhar et al. (1989);


(3) \begin{equation*} {C}_i/{C}_a=\frac{\left({\varDelta}^{13}C-a\right)}{\left(b-a\right)} \end{equation*}

Thus, we estimate iWUE, a measure of the amount of carbon assimilated per unit leaf area per unit time per unit cost of water using *C_i_* and *C_a_* according to (Ehleringer 1993):


(4) \begin{equation*} iWUE=\frac{A}{g_s}=\frac{\left({C}_a-{C}_i\right)}{1.6}={C}_a\times \frac{1-\frac{C_i}{C_a}}{1.6}=\frac{C_a\left(b-{\varDelta}^{13}C\right)}{1.6\left(b-a\right)} \end{equation*}

Where *1.6* is the ratio of diffusivities of water and CO_2_ in air.

The observed iWUE values for our study sites computed using Eq. 4 were then compared with three theoretical scenarios for the regulation of plant-gas exchange fractionation during CO_2_ diffusion through the stomata, as outlined by Saurer et al. (2004). In scenario 1, C_i_ remains constant as C_a_ increases, resulting in a reduction in the C_i_/C_a_ ratio due to strong stomatal closure. Scenario 2 reflects a proportional regulation of C_i_ by photosynthesis and stomatal conductance, resulting in a constant C_i_/C_a_ ratio. In scenario 3, C_i_ exactly follows the increase in C_a_, representing a relatively weak stomatal response that results in a constant difference between C_a_ and C_i_.

### Estimates derived from oxygen isotope values

We estimated the ^18^O enrichment of leaf water, by first calculating tree-ring cellulose enrichment above source water (Δ^18^O_ring_) between 1990 to 2022 from raw tree ring δ^18^O (δ^18^O_ring_) and assuming no fractionation during root water uptake.


(5) \begin{equation*} \triangle^{18}{\textrm O}_{\textrm ring} = \frac{\delta^{18}{O}_{ring}-{\delta}^{18}{O}_{prec}}{1+\left(\frac{\delta^{18}{O}_{prec}}{1000}\right)} \end{equation*}

Where *δ^18^O*_*pre*c_ is the oxygen isotopic composition of precipitation the year of ring formation. Given the lack of long-term records of *δ^18^O_prec_* at the study locations, we estimated site-specific δ^18^O_prec_ over 1990–2022 following Barbour et al. (2002):


(6) \begin{align*} {\delta}^{18}{O}_{prec}=\,&0.52\ast{T}_a-0.006\ast{\left({T}_a\right)}^2+2.42\ast{P}_a\nonumber\\&-1.43\ast{\left({P}_a\right)}^2-0.046\ast \sqrt{E}-13 \end{align*}

T_a_ is the mean annual temperature (in degrees Celsius), P_a_ is the total annual precipitation (in meters), obtained from ERA5 (Copernicus Climate Change Service 2024) and E is the site elevation (in meters a.s.l.). To assess the reliability of precipitation δ^18^O values estimated using Eq. 6, we extracted monthly precipitation δ^18^O data from the closest station of the Global Network of Isotopes in Precipitation network (GNIP; Schotterer et al. 1996). This station was Chapais (49.82° N–74.97° W) and we converted the monthly precipitation δ^18^O data into an annual time series spanning 1997–2010 by calculating the average of the monthly δ^18^O values for each year. This approach provides a representative annual δ^18^O value based on the available monthly data. Comparison between the two datasets was done using a scatterplot and Pearson’s correlation coefficient (Figure S1).

Finally, from Δ^18^O_ring_ we calculated the enrichment of leaf water ^18^O above source water (Δ^18^O_lw_) using the following:


(7) \begin{equation*} {\varDelta}^{18}{O}_{lw}=\frac{\varDelta^{18}{O}_{ring}-\varepsilon wc}{1-{P}_x{P}_{ex}} \end{equation*}

Where, ε_wc_ is the temperature-dependent fractionation associated with carbonyl oxygen atoms exchanging with water during wood (i.e. cellulose) synthesis (Cheesman & Cernusak 2017; Sternberg et al. 1986), p_x_ is the fraction of cambial cellular water that is not isotopically fractionated (px ≈ 1.0), and p_ex_ is the fraction of carbonyl oxygen atoms that exchange with non-fractionated water (Barbour & Farquhar, 2000). Generally, a fixed value of 0.4 is considered for p_x_p_ex_ (Guerrieri et al. 2019; Mathias & Thomas, 2021; Palandrani et al. 2021).

We calculate ε_wc_ following Sternberg & Ellsworth (2011) using the following:


(8) \begin{equation*} {\varepsilon}_{wc}=0.0084\ast{T}_a^2-0.51\ast{T}_a+33.172 \end{equation*}

### Climatic data

We obtained climatic data from ERA5 (ECMWF’s fifth-generation atmospheric global climate reanalysis; Bell et al. 2021; Hersbach et al. 2020). The data were accessed from the Copernicus Climate Change Service (2024). The climatic data were retrieved from the lowest atmospheric level (surface level) of each ERA5 grid cell that encompassed our study sites. We obtained four ERA5 datasets consisting of monthly values of 2-m air temperature (°C), 2-m dewpoint temperature (°C), surface pressure (kPa) and precipitation (mm). To calculate VPD, we used the plantecophys package (Duursma 2015) in R version 4.2.2 (R Core Team 2020). Finally, using the retrieved data, we calculated the growing season (April to September) mean temperature (T_grs_), total precipitation (P_grs_) and vapor pressure deficit (VPD_grs_), respectively.

### Data analysis

We calculated Pearson’s correlation coefficient (r) to assess the relationships between Δ^18^O_lw_ and observed climate variables (growing season temperature, precipitation and VPD) from 1990 to 2022 at the three study sites. We employed linear regression to analyze the relationships between iWUE and C_a_, and between BAI and iWUE. Temporal trends over the study period were evaluated using regression analysis. Furthermore, we applied multiple linear regression (MLR) to determine how growing season climate variables, C_a_, tree age and study sites influence long-term iWUE. Similarly, MLR was used to examine how these factors, along with iWUE, impact long-term BAI in the study sites. To identify the most influential predictors for iWUE and BAI, we used a bidirectional stepwise regression approach, iteratively adding or removing variables from the full model to identify key predictors. After determining these predictors, we developed four different models for each response variable (Table S3). Lastly, we compared these models using the Akaike Information Criterion corrected for small sample sizes (AICc) to select the best-fitting model for our data.

## Result

### Temporal trends in carbon and oxygen isotope-derived variables δ^13^C, Δ^13^C and ΔO^18^_lw_

**Figure 3:**
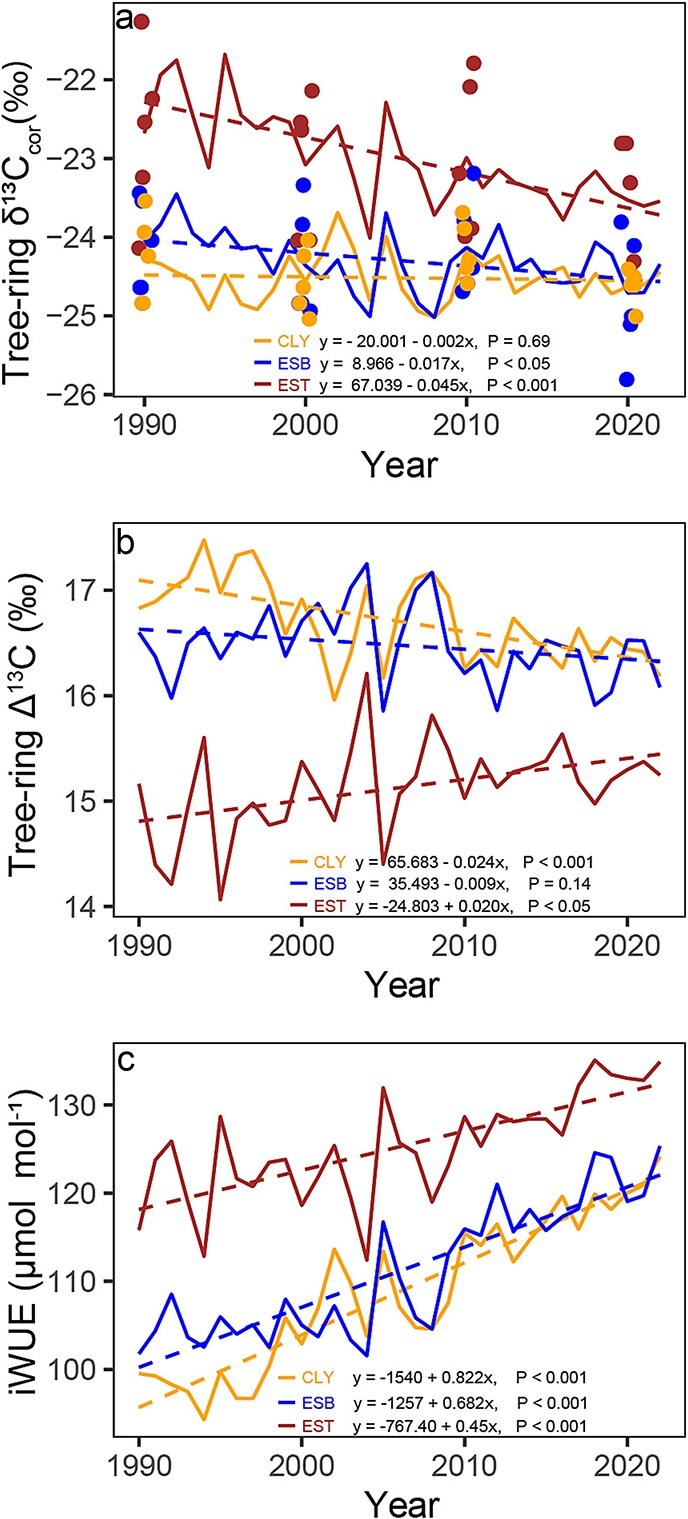
Temporal variation of carbon isotope-derived variables for *P. banksiana* from 1990 to 2022 across the study sites. (a) Tree-ring carbon isotope ratio (δ^13^C_cor_). Each dot represents an individual tree measurement, while the chronologies are composite values derived from five trees. Points are jittered along the x-axis to prevent overlap. (b) Tree-ring carbon isotope discrimination (Δ^13^C) across the study sites. (c) Temporal variations in iWUE across the study sites from 1990 to 2022. Regression lines in this figure illustrate trends over time.

**Figure 4:**
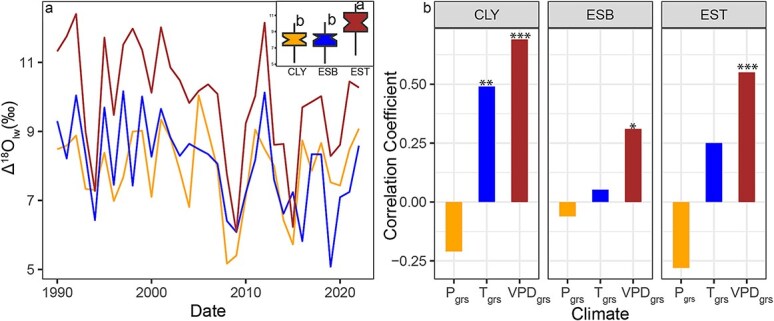
(a) Temporal changes in leaf water enrichment (Δ^18^O_lw_) for *P. banksiana* from 1990 to 2022 across the study sites. The boxplot inset shows the distribution of Δ^18^O_lw_ values per site across the study period. Different letters indicate significant differences (*P* < 0.001) between groups based on pairwise t-tests (b) correlation between average climatic variables during the growing season (April to September) and leaf water enrichment (Δ^18^O_lw_) across the study sites, with ^*^ indicating significance at *P* < 0.05, ^**^ significance at *P* < 0.01 and ^***^ significance at *P* < 0.001. P_grs_ denotes growing season precipitation, T_grs_ represents growing season temperature and VPD_grs_ signifies growing season vapour pressure deficit.

δ^13^C_cor_ decreased since 1990 for *P. banksiana* at EST (slope = −0.045 ‰/year, *P* < 0.001) and ESB site (slope = −0.017‰/year, *P* < 0.05), even after correcting to a pre-industrial standard (Figure 3a). By contrast, no significant trend was detected at the CLY site (slope = −0.002‰/year, *P* = 0.69). The decline in δ^13^C_cor_ was more pronounced at the EST site than at ESB. The mean δ^13^C_cor_ ​value at EST was −23.0‰, compared with −24.30‰ at ESB and − 24.52‰ at CLY. This indicate that the more negative δ^13^C_cor_ ​values at ESB and CLY indicate higher carbon discrimination (Δ^13^C) than at EST (Figure 3b). The dispersion of δ^13^C_cor_ values measured from individual tree in 1990, 2000, 2010 and 2020, varies across years and sites. At the EB site, values range from −24.06 ± 0.58‰ in 1990 to −24.77 ± 0.81‰ in 2020. At the EST site, they range from −22.68 ± 1.09‰ in 1990 to −23.53 ± 0.79‰ in 2020, while at the ANT site, they range from −24.28 ± 0.57‰ in 1990 to −24.63 ± 0.23‰ in 2020. The ΔO^18^_lw_ ​ values derived from tree-ring δ^18^O had mean values of 9.93‰, 7.93‰ and 8.07‰ at the EST, CLY and ESB sites, respectively. Pairwise t-tests further revealed that ΔO^18^_lw_ at the EST was significantly different from CLY and ESB (*P* < 0.001) (Figure 4a).

### Long-term changes in iWUE and the effects of environmental variables on iWUE and ΔO^18^_lw_

We observe an increasing trend in the iWUE of *P. banksiana* at all study sites: EST (slope = 0.45 μmol mol^−1^ year^−1^, *P* < 0.001), ESB (slope = 0.682 μmol mol^−1^ year^−1^, *P* < 0.001) and CLY (slope = 0.822 μmol mol^−1^ year^−1^, *P* < 0.001) (Figure 3c). The mean iWUE ​value at EST was 125.26 μmol mol^−1^, compared with 111.14 μmol mol^−1^ at ESB and 108.82 μmol mol^−1^ at CLY. A multiple linear model (Table 1) explains 90% of the total variance in iWUE at our study sites from 1990 to 2022. The model reveals a significant positive relationship between iWUE, atmospheric carbon (C_a_) and growing season vapor pressure deficit (VPD_grs_). The model also indicates that the effect of C_a_ on iWUE varies by site, C_a_’s effect on iWUE being weaker at EST site compared with CLY (baseline site; *P* < 0.001). Additionally, significant differences in iWUE were observed among sites, being higher at EST site than CLY site (baseline site; *P* < 0.001), while no significant difference was found between CLY and ESB (*P* = 0.11; Table 1). Furthermore, we observed that ΔO^18^_lw_ showed a significant positive correlation with VPD_grs_ at all sites and growing season temperature only at CLY (T_grs_​; *r* = 0.49, *P* < 0.01) (Figure 4b and Table S4). Additional correlation analyses between ecophysiological and environmental variables, as well as correlation coefficients among meteorological variables, are provided in Tables S4 and S5, respectively.

**Table 1:** Multiple linear regression for iWUE. The parameters included in the model are VPD_grs_ during the growing season (April to September), atmospheric C_a_ and site location. The R-squared value of the model is 0.90. The reference site is the Clay site (CLY). Site effects are to be interpreted relative to the intercept, whereas interaction terms represent differences in slope relative to CLY for the relationship with Ca. Model selection can be found in Table S3.

	Estimate	Std. error	t-value	p- value
**Intercept**	−65.12	12.00	−5.43	< 0.001
**C**_**a**_	0.42	0.03	13.66	< 0.001
**VPD**_**grs**_	33.78	6.59	5.12	< 0.001
**SiteEsker_Base**	26.62	16.41	1.62	0.11
**SiteEsker_Top**	86.63	16.41	5.28	< 0.001
**C**_**a**_ **:SiteEsker_Base**	−0.07	0.043	−1.51	0.14
**C**_**a**_ **:SiteEsker_Top**	−0.19	0.04	−4.31	< 0.001

The three proposed gas exchange scenarios, used as a baseline for interpreting gas exchange responses to rising atmospheric C_a_, are presented in Figure S2. The variation in iWUE from 1990 to 2022 across the three sites (EST, ESB and CLY) aligned most closely with scenario 2, where C_i_/C_a_ remained constant. However, given the relatively short time span of our site-specific data, it is premature to conclude that scenario 2 definitively governs the observed trends.

### Long-term changes in BAI and the effect of environmental variables on BAI

**Figure 5:**
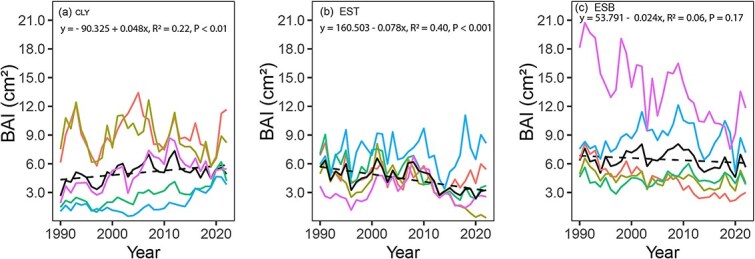
Changes in BAI across the study sites. The trend in BAI is shown for five trees (each representing the average of two radii), with a linear regression line fitted to illustrate the trend.

The BAI of *P. banksiana* at the CLY site increased over the last 33 years, with a slope of 0.048 cm_2_ year_−1_ (*P* < 0.01) (Figure 5a). In contrast, a decline in BAI was observed at the EST site, with a slope of −0.078 cm_2_ year_−1_ (*P* < 0.001) (Figure 5b). No significant trend was detected at the ESB site (Figure 5c). A multiple linear model (Table 2) explains 66% of the total variance in BAI at our study sites from 1990 to 2022. The results indicate a significant positive relationship between BAI and both growing season precipitation (P_grs_) and vapor pressure deficit (VPD_grs_). iWUE is almost significant (*P* = 0.09). Furthermore, tree growth varies significantly across sites. Additionally, we observe a significant negative interaction between iWUE and site, with the strongest negative interaction occurring at Esker Top (EST), suggesting that, despite increases in iWUE, reduced tree growth is observed at this site.

**Table 2:** Linear model for BAI. The parameters included in the model are precipitation (P_grs_), VPD_grs_ during the growing season (April to September), iWUE, tree age and site characteristics. The R-squared value of the model is 0.66. The reference site is the Clay site (CLY). Site effects are to be interpreted relative to the intercept, whereas interaction terms represent differences in slope relative to CLY for the relationship with iWUE. Model selection can be found in Table S3.

	Estimate	Std. error	t-value	*p*- value
**Intercept**	−8.719	2.259	−3.86	<0.001
**P**_**grs**_	0.042	0.010	4.09	<0.001
**VPD**_**grs**_	12.464	2.024	6.16	<0.001
**Tree_Age**	−0.003	0.016	−0.18	0.85
**iWUE**	0.41	0.024	1.72	0.09
**SiteEsker_Base**	10.510	2.855	3.68	<0.001
**SiteEsker_Top**	21.065	3.503	6.01	<0.001
**SiteEsker_Base: iWUE**	−0.087	0.024	−3.58	< 0.001
**SiteEsker_Top: iWUE**	−0.181	0.028	−6.43	< 0.001

### Relationships between iWUE and BAI

**Figure 6:**
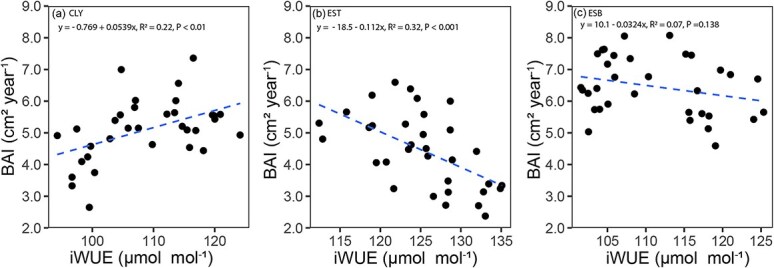
Relationships between BAI and iWUE across the three study sites, with a linear regression line fitted to illustrate the trend.

The influence of gas exchange on trees’ BAI was also examined. Despite a general positive effect of iWUE (Table 2), BAI increases significantly with increasing iWUE only at CLY site (R^2^ = 0.22, *P* < 0.01). Conversely, at EST site BAI decreases significantly with increasing iWUE (R^2^ = 0.32, *P* < 0.001), and no relationship is found at ESB site (R^2^ = 0.07, *P* = 0.14) (Figure 6a–c).

## Discussion

### Strong stomatal regulation of P. banksiana, especially at drier sites

Our study revealed a significant correlation between Δ^18^O_lw_ and VPD_grs_ in *P. banksiana*, highlighting the dominant role of g_s_ in regulating the species’ physiological response to atmospheric conditions (Figure 4b and Table S4). VPD_grs_ is a key driver of plant water relations, directly influencing stomatal behavior and thus transpiration rates. The observed positive correlation suggests that under high evaporative demand, jack pine close their stomata to conserve water, leading to prolonged leaf water residence time and increased δ^18^O enrichment due to evaporative fractionation (Gessler et al. 2014; Guerrieri et al. 2019). Additionally, the observed correlation between Δ^18^O_lw_ and VPD_grs_ could indicate that high evaporative demand at the study site may have promoted kinetic fractionation in the soil water, potentially leading trees to access isotopically enriched water. However, the absence of long-term soil water isotopic data precluded us from testing this hypothesis. Therefore, we propose that the stomatal closure observed in trees at our study sites reflects an adaptive regulation mechanism that optimizes carbon assimilation while minimizing water loss under fluctuating moisture conditions. This dominance of stomatal control aligns with the well-documented water-conservation strategy of *P. banksiana*, which promptly responds to atmospheric dryness by adjusting g_s_ (Dang et al. 1997).

Our result further highlights that while g_s_ serves as an important physiological mechanism regulating *P. banksiana* across our study sites, its influence is notably stronger at the drier EST site compared with ESB and CLY, as indicated by less negative δ^13^C values and higher Δ^18^O_lw_ (Figure 3a and  4a). This pronounced stomatal response at EST is likely driven not only by an adaptive reaction to higher evaporative demand but also by site-specific characteristics unique to EST, which further reinforce this strong stomatal regulation. The EST site, located at the top of the esker ridge, is relatively drier and features sandy soils with rapid drainage, low water retention and limited soil moisture availability (Menzies et al. 2018). These conditions create an environment of increased water stress, necessitating more conservative water use by the trees. The combination of high VPD and poor soil water retention likely amplifies the need for trees at EST to adjust their stomatal behavior to cope with increased evaporative demand and constrained water supply. Our findings at this site further highlight the pivotal role of hydroclimatic conditions in shaping the physiological responses of *P. banksiana* in the boreal forest. At EST, trees seem to adopt a water-efficient but carbon-limited strategy to survive in dry conditions. However, prolonged stomatal closure may eventually limit carbon assimilation and hinder growth, especially during prolonged droughts or rising vapor pressure deficits (Grossiord et al. 2020).

Furthermore, we observed a significant increase in iWUE across our study sites, as evidenced by the combined effect of elevated atmospheric C_a_ and VPD_grs_. The increase in iWUE suggest that *P. banksiana* optimizes its photosynthetic performance while reducing water loss under changing environmental condition. Increasing C_a_ likely promotes carbon uptake by increasing the CO_2_ gradient between atmosphere and leaf intercellular spaces, thereby allowing the species to maintain assimilation rates with less stomata opening. Concurrently, higher VPD_grs_ suggests that increasing evaporative demand, appears to trigger partial stomata closure as a water conversation measure thus further amplifying iWUE (Grossiord et al. 2020). This interaction underscores the physiological plasticity of *P. banksiana*, allowing it to maintain productivity and resilience within the boreal ecosystem despite rising atmospheric CO_2_ and evolving climatic stressors such as VPD. Our findings are consistent with previous studies with other species reporting increased iWUE as a consequence of elevated C_a_ and rising VPD conditions (Andreu-Hayles et al. 2011; Peñuelas et al. 2011; Pu & Lyu, 2022).

### Increased iWUE may come at the cost of reduced tree growth at drier site

*Pinus banksiana* in the boreal forests of western northern Quebec exhibits remarkable ecological adaptability, thriving across diverse environments, from rocky outcrops and nutrient-poor sandy eskers to mesic, clay-rich soils (Bergeron & Bouchard, 1984). Its dominance at the EST site is likely due to its ability to establish in well-drained, coarse-textured soils where water availability is limited. Notably, *P. banksiana* growth across our study sites is positively associated with growing seasons characterized by both higher precipitation and greater atmospheric dryness, suggesting an ability to balance carbon assimilation and water conservation under drier growing seasons conditions.

However, our results reveal a complex interplay between iWUE and BAI in *P. banksiana* particularly at the drier EST site, where increased iWUE does not translate into enhanced growth. This finding is evidenced by a negative slope in the iWUE–BAI relationship and further reinforced by less negative δ^13^C values compared with other sites. This observed pattern suggests that *P. banksiana* at EST site is under considerable water stress, which prompts more frequent or prolonged stomatal closure to reduce water loss. While this regulation conserves water, it also limits CO_2_ uptake, thereby limiting *A* (Farquhar & Sharkey, 1982; Pirasteh-Anosheh et al. 2016). The decline in *A*, in turn, reduces the availability of carbon needed for biomass accumulation, ultimately resulting in lower BAI. The trade-off between water conservation and carbon assimilation highlights a key limitation in the drought response strategy of *P. banksiana* at dry sites. Although increased iWUE reflects a shift toward more conservative water use, it appears insufficient to sustain growth under prolonged water deficits (Aranda et al. 2024; Guo et al. 2024). These findings emphasize that under increasingly dry conditions, physiological adjustments alone may not fully offset the negative impacts of water stress on growth, pointing to potential long-term vulnerabilities for *P. banksiana* populations in such environments (Allen et al. 2015; Seleiman et al. 2021; Song et al. 2022).

Furthermore, this decrease in BAI might also result from shifts in carbon allocation strategy over time. In water-stressed environments like EST site, trees might prioritize carbon allocation towards root development particularly fine roots to improve water uptake and enhance survival, rather than to stem growth (Giguère-Croteau et al. 2019; C. Liu et al. 2023). Given that *P. banksiana* is known for its extensive root system (Burns 1990; Cissé et al. 2025), this shift in allocation may explain the observed decoupling of iWUE and BAI. Additionally, the decline in tree growth observed at the EST site may also be attributed to non-photosynthetic limitations, such as reduced cell turgor pressure and constrained nutrient uptake in dry, nutrient-poor soils, rather than solely to carbon limitation (Körner 2003). Turgor pressure, which is generated through water uptake from the soil, is essential for cell expansion and wood formation (Rossi et al. 2009). In drier environment with elevated VPD and low soil moisture, as observed at the EST site, available water for cell turgor is reduced, leading to a decline in cell enlargement and division, ultimately limiting radial growth (Marchand et al. 2021; Novick et al. 2024). This mechanism has been demonstrated in *P. banksiana*, where reduced turgor pressure under drought conditions has been associated with diminished stem growth (Blake & Li, 2003; Marshall et al. 2000). Moreover, low soil moisture also hampers nutrient mobility and uptake, further exacerbating growth limitations. The EST site’s sandy texture, low water-holding capacity, and inherently poor nutrient status likely intensify these stressors. These findings are consistent with previous study showing that tree growth declines are more severe in drought-prone, nutrient-limited environments (Lévesque et al. 2016). Therefore, the growth reduction observed at EST not only results from direct limitation of photosynthetic carbon supply but also from a combination of reduced turgor-driven cell expansion and slower nutrient acquisition constraints. Thus, our study suggests that in water-limited regions of the boreal forest, *P. banksiana* might experience a decline in BAI despite an increase in iWUE.

At the CLY site, we observed that an increase in iWUE translates to a corresponding increase in BAI. These findings suggest that the efficient water use of *P. banksiana* at CLY is combined with enhanced carbon assimilation and biomass accumulation. The CLY site, characterized by its humid conditions and proximity to groundwater during the growing season (Cloutier et al. 2015), provides favorable moisture conditions that support continuous water uptake. As a result, trees at this site are likely able to maintain sufficient turgor pressure, which facilitates not only cell expansion but also active cell division, a key process that supports this observed growth pattern. Moreover, in the study region, clay deposits are generally more fertile than coarser glacial deposits, providing greater nutrient availability (Légaré et al. 2001). This combination of adequate water and nutrient supply may have further supported the observed increase in basal area growth, reinforcing the positive relationship observed between iWUE and BAI at CLY. Several studies conducted in similar ecological conditions have demonstrated that clay-rich soils tend to be more nutrient-dense and promote greater forest productivity (Béland & Bergeron, 1996; Royer-Tardif & Bradley, 2011; Ste-Marie et al. 2007). In contrast, at the ESB site, the relationship between iWUE and BAI is not significant (Figure 6c). This could be due to ESB’s intermediate environmental conditions between EST and CLY, possessing sandy soil characteristics similar to EST while also exhibiting some humid site traits like CLY.

### Predicting critical impacts of climate change on P. banksiana physiology and growth

*Pinus banksiana*, the most widely distributed pine in Canada and a vital resource for the pulp and lumber industries (Law & Valade, 1994), could face significant threats from climate change in the boreal forest. With mean annual temperatures already rising by 1.7 °C over the past century and a further increase of 2 °C observed recently, projections suggest an additional rise of up to 6 °C by the century’s end (Price et al. 2013). While some regions may experience increased spring and early summer rainfall, this is unlikely to offset the higher evapotranspiration rates driven by elevated temperatures and VPD, particularly in drier, coarse-textured sites (Johnston 2009; Vincent et al. 2018). Under increased drought stress at drier sites, *P. banksiana* may close its stomata to conserve water. However, this can reduce photosynthetic carbon assimilation and increase maintenance respiration, ultimately leading to reduced growth, as observed at the EST site. The patterns observed in our study at EST site suggest that these predicted climate-induced stresses are already beginning to manifest, reinforcing concerns about the species’ resilience in drier environments.

Despite these challenges, our results suggest that the impact of climate change on *P. banksiana* will not be entirely negative. Rising atmospheric CO_2_ levels associated with climate change will enhance iWUE at favorable sites and increase growth, provided that water and nutrient availability are not limiting factors (Johnston, 2009). This is evident at the CLY site, where fertile clay soils and adequate moisture support *P. banksiana* growth despite rising temperatures and high stomatal regulations. *Pinus banksiana* may even enhance its growth performance into such areas over the coming decades, except where soils are waterlogged (Genries et al. 2012).

Collectively, our findings suggest that *P. banksiana* may face divergent trajectories: reduced growth and productivity in drier sites like EST, sustained or enhanced growth in humid areas like CLY, and unpredictable responses in intermediate zones like ESB. This underscores the need for site-specific considerations when interpreting forest productivity trends in the context of ongoing climate change within the boreal forest. This finding has important implications for the modeling of vegetation response to atmospheric variability. Most ecophysiological and dynamic global vegetation models predict an increase in plant biomass due to a stimulation of photosynthesis by C_a_ (Sitch et al. 2008). Given the results presented here, such models might overestimate future forest carbon storage capacities at drier sites such as EST. It is important to interpret our results with caution when extrapolating them to other parts of the boreal forest, as the study has certain limitations in terms of sample size. Additionally, other physiological mechanisms such as mesophyll conductance and Rubisco activity which were beyond the scope of this study, may also play roles in regulating *P. banksiana* growth across our study sites (Beauclaire et al. 2024; Flexas et al. 2008). Furthermore, factors like genetic variability and post-assimilation ^13^C fractionation, also not assessed here, could potentially contribute to differences observed among sites (Gessler et al. 2014, Bartholomé et al. 2015). Despite these limitations, the comprehensive analysis of *P. banksiana* growth and physiology presented in this study provides valuable insights into the mechanisms by which the species may respond to ongoing climate change, offering a foundation for future research and forest management strategies.

## Supplementary Material

Olugbadieye_et_al_Supplimentary_tpaf102

## Data Availability

Available upon request.
