# Supplementary material for: Jack pine’s responses to climate change: increased water–use efficiency but evident growth limitations in dry environments
Source: Tree Physiol. 2025 Aug 22;45(9):tpaf102. doi: 10.1093/treephys/tpaf102 (PMC12482914; doi:10.1093/treephys/tpaf102)
Supplement: Olugbadieye_et_al_Supplimentary_tpaf102 [file olugbadieye_et_al_supplimentary_tpaf102.docx]

**Supplementary tables**

Table S1: Summarized information on the study sites. Approximate mean water table depth during summer was obtained from the nearby points of a map derived from interpolation of piezometric data; Cloutier et al., 2015). Climate variables were obtained from the Copernicus Climate Change Service (2024).

| Sites | Latitude (^o^N) | Longitude (^o^W) | Elevation (m) | Soil water depth (m) | Mean growing season temperature (^o^C) | Mean growing season precipitation (mm) |
| --- | --- | --- | --- | --- | --- | --- |
| CLY | 49.15 | 78.80 | 311 | 0-5 | 11.5 | 544.7 |
| ESB | 48.52 | 78.21 | 324 | 5-10 | 12.1 | 557.6 |
| EST | 48.53 | 78.21 | 342 | 20-25 | 12.1 | 557.6 |

Table S2. Characteristics of the cross-dated tree-ring width series

| Parameter | Authier Nord  (CLY) | Esker Base  (ESB) | Esker Top  (EST) |
| --- | --- | --- | --- |
| Chronology time span | 1924-2022 | 1977-2022 | 1944-2022 |
| Number of tree-rings at breast height | 91 - 99 | 36-46 | 54-79 |
| Number of trees | 5 | 5 | 5 |
| Mean series intercorrelation ± SD | 0.57 ± 0.09 | 0.56 ± 0.14 | 0.59 ± 0.10 |
| Mean BAI over 1990-2022 (cm^2^) ± SD | 5.101± 0.984 | 6.459 ± 0.931 | 4.443 ± 1.188 |
| Mean BAI before 1990 (cm^2^) ± SD | 3.201±0.925 | 3.771±2.257 | 5.134±1.993 |
| Mean sensitivity | 0.20 | 0.19 | 0.26 |
| First order autocorrelation (Ar1) | 0.91 | 0.91 | 0.86 |
| Gini | 0.32 | 0.28 | 0.32 |
| Expressed Population Signal (EPS) | 0.93 | 0.99 | 0.97 |

Table S3: Summary of multiple linear models developed for intrinsic water-use efficiency and basal area increment across the three study sites. The models incorporate key predictors, including climatic variables (e.g., growing season precipitation, vapor pressure deficit), atmospheric CO₂ concentrations, tree age, and site-specific effects, to identify factors influencing iWUE and BAI over time. We proceed with model selection by evaluating multiple candidate models using Akaike’s Information Criterion corrected for small sample sizes (AICc), which balances model fit and complexity. The model with the lowest AICc is considered the best-supported, while the difference in AICc values (ΔAICc) helps determine the relative strength of each model. In this study, models with ΔAICc = 0 have substantial support, while those with higher values are less plausible.

| Response Variables | Models | K | AICc | ΔAICc | AICc Weight |
| --- | --- | --- | --- | --- | --- |
| iWUE | Ca + VPD_grs_ + Ca*Site  (Ca + VPD_grs_) *Site  Ca + VPD_grs_ + Tree Age + Site  Ca + VPD_grs_ *Site | 8  10  7  8 | 527.56  531.53  536.22  545.38 | 0.00  3.97  8.66  17.82 | 0.87  0.12  0.01  0.00 |
| BAI | Prec_grs_ + VPD_grs_ + Tree Age + IWUE*Site  (Prec_grs_ + VPD_grs_ + Tree Age + IWUE) *Site  (Prec_grs_ + VPD_grs_ + IWUE) *Site  (Prec_grs_ + VPD_grs_) *Site | 10  16  13  10 | 700.41  701.57  705.19  744.09 | 0.00  1.16  4.77  43.67 | 0.60  0.34  0.06  0.00 |

Table S4: Pearson correlation coefficients between ecophysiological variables and growing season temperature (Tgrs), precipitation (Pgrs), and vapor pressure deficit (VPDgrs) across the three study sites. Statistical significance is indicated by asterisks at p< 0.05, ** significance at p< 0.01 and *** significance at p < 0.001

|  | Authier Nord (CLY) | | | | Esker top (EST) | | | | Esker base (ESB) | | | |
| --- | --- | --- | --- | --- | --- | --- | --- | --- | --- | --- | --- | --- |
|  | δ¹³C_cor_ | Δ^13^C | iWUE | Δ¹⁸O_lw_ | δ¹³C_cor_ | Δ^13^C | iWUE | Δ¹⁸O_lw_ | δ¹³C_cor_ | Δ^13^C | iWUE | Δ¹⁸O_lw_ |
| T_grs_ | 0.46** | -0.54** | 0.41* | 0.49** | -0.02 | -0.13 | 0.37* | 0.16 | -0.03 | -0.16 | 0.27 | 0.06 |
| P_grs_ | -0.16 | 0.09 | -0.02 | -0.25 | -0.27 | 0.27 | -0.03 | -0.33* | -0.21 | 0.10 | 0.08 | -0.13 |
| VPD_grs_ | 0.59** | -0.47** | 0.22 | 0.67*** | 0.33* | -0.44* | 0.32* | 0.52** | 0.32* | -0.33* | 0.15 | 0.36* |

Table S5: Pearson correlation coefficients among meteorological variables growing season temperature (Tgrs), precipitation (Pgrs), and vapor pressure deficit (VPDgrs) for the three study sites. Statistical significance is indicated by asterisks at p< 0.05, ** significance at p< 0.01 and *** significance at p < 0.001.

|  | Authier Nord | | | Esker top | | | Esker base | | |
| --- | --- | --- | --- | --- | --- | --- | --- | --- | --- |
|  | T_grs_ | P_grs_ | VPD_grs_ | T_grs_ | P_grs_ | VPD_grs_ | T_grs_ | P_grs_ | VPD_grs_ |
| T_grs_ |  | -0.32* | 0.76*** |  | -0.27 | 0.77*** |  | -0.27 | 0.77*** |
| P_grs_ | -0.32* |  |  | -0.27 |  |  | -0.27 |  |  |
| VPD_grs_ | 0.76*** | -0.53** |  | 0.77*** | -0.55*** |  | 0.77*** | -0.55*** |  |


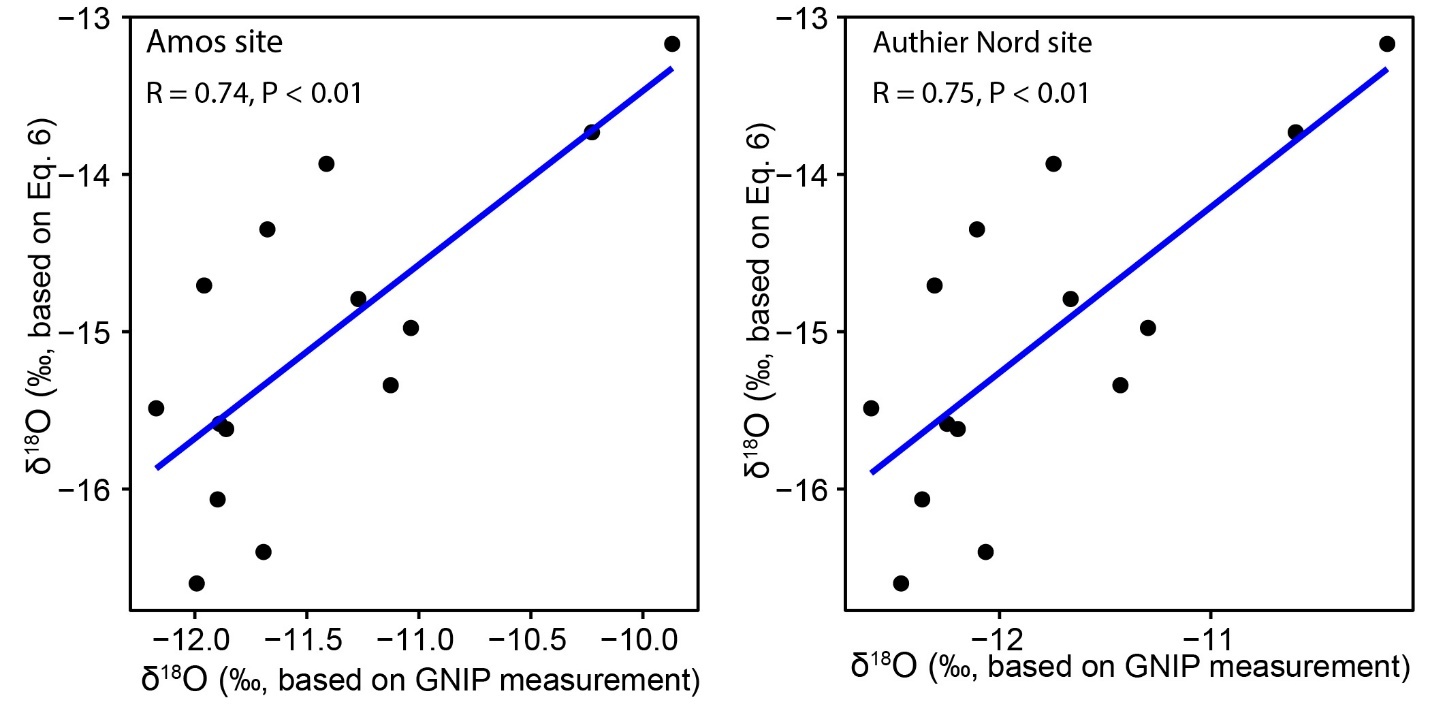
Figure S1: Correlation between GNIP δ^18^O precipitation and δ^18^O value estimated using equation 6.


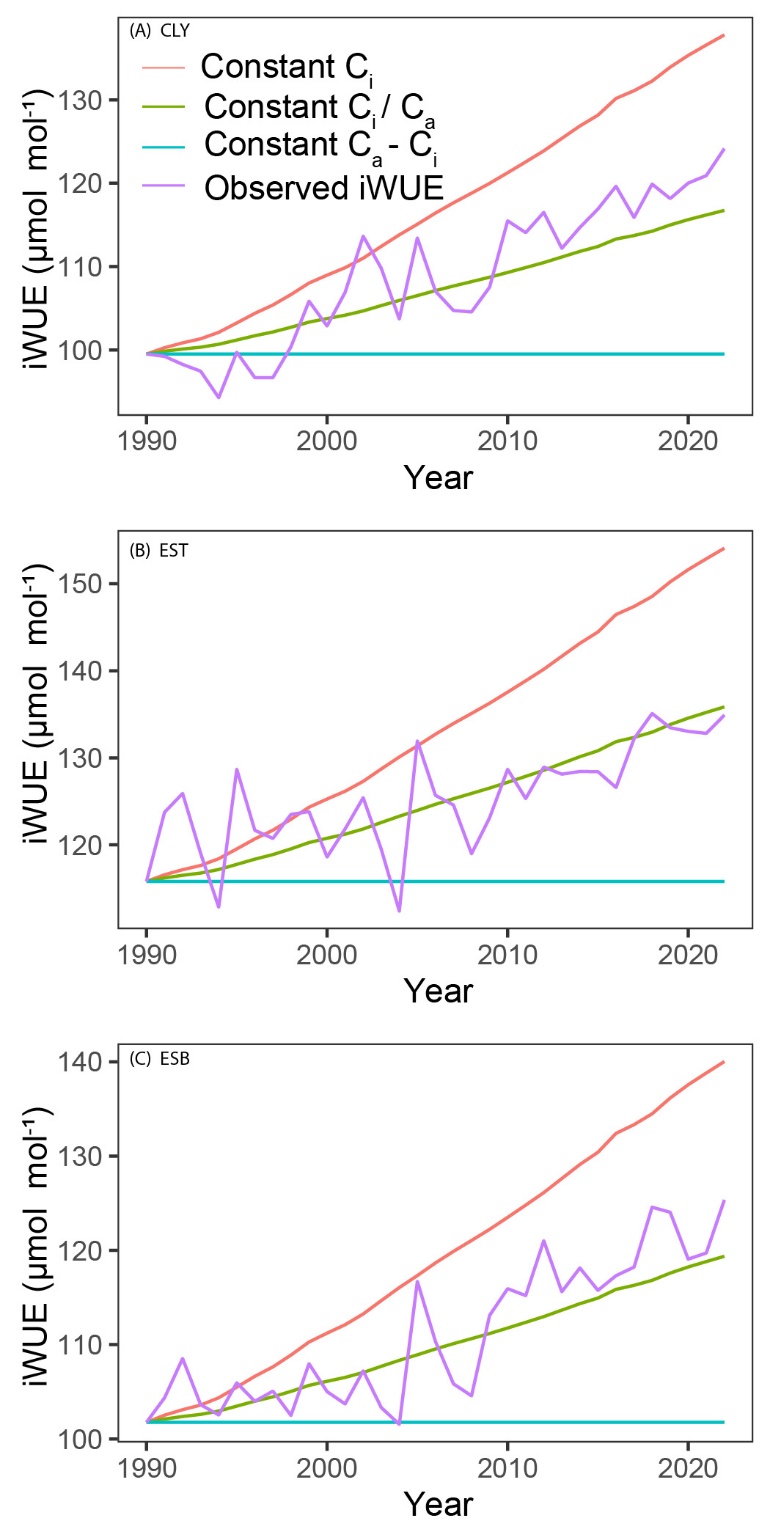


Figure S2: Observed intrinsic water-use efficiency (iWUE) at three study sites and iWUE calculated under three scenarios serving as a baseline for interpreting gas exchange responses to rising C_a_.


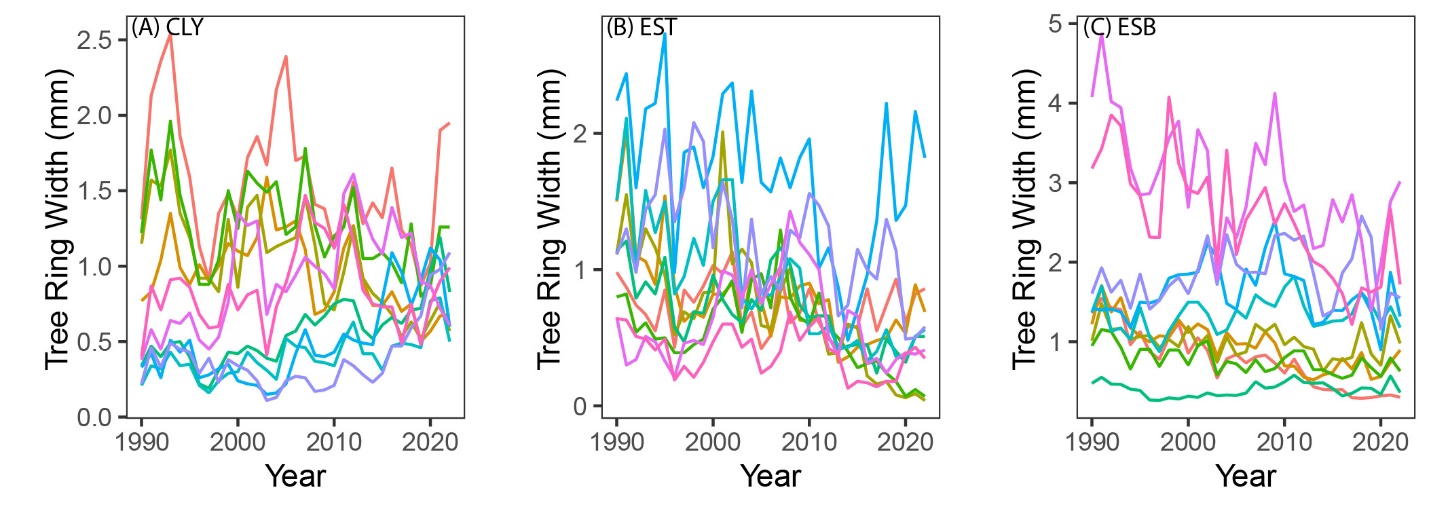


Figure S3: Raw tree-ring width measurements (mm) for each core extracted from five sampled *P. banksiana* trees at the three study sites: (A) CLY (humid site), (B) EST (dry site), and (C) ESB (intermediate site). Each colored line represents an individual core.
